# Efficacy of rotator cuff suture and arthroscopic 360° capsular release in patients with rotator cuff tear with limited shoulder movement

**DOI:** 10.1186/s12893-023-02157-6

**Published:** 2023-12-13

**Authors:** Jianwei Zuo, Chen Chen, Jiang Guo, Jianjing Lin, Tian You, Peng Chen, Canfeng Li, Wei Li

**Affiliations:** 1 Department of Sports Medicine, Peking University Shenzhen Hospital, 1120 Lianhua Road, Shenzhen, Guangdong 518036 P.R. China; 2 Department of Anesthesiology, Peking University Shenzhen Hospital, 1120 Lianhua Road, Shenzhen, Guangdong 518036 P.R. China

**Keywords:** Rotation cuff injury, Arthroscopy, Shoulder joint, Joint capsule release

## Abstract

**Background:**

To determine the clinical efficacy of rotator cuff suture and arthroscopic 360° capsular release in patients with rotator cuff tendinopathy to improve the Constant-Murley and Visual Analogue Scale (VAS) scores, and shoulder flexion.

**Methods:**

Fifty-one patients with full-thickness rotator cuff tears and limited shoulder movement who were admitted to our hospital from October 2017 to October 2020 were selected; all patients were treated with arthroscopic rotator cuff suture and 360° capsular release. The Constant-Murley score, VAS score, and shoulder flexion angle were used to evaluate shoulder joint function before and during follow-up. Rotator cuff healing was assessed by MRI with the Sugaya classification.

**Results:**

After treatment, the Constant-Murley score (58.98 ± 9.84) was significantly improved compared with pre-treatment (29.33 ± 9.71), the VAS score (1.23 ± 0.87) was significantly lower than pre-treatment (7.54 ± 1.22), and the shoulder flexion angle (142.67 ± 8.59°) was significantly improved compared with pre-treatment (51.50 ± 2.10°); the difference was statistically significant (P < 0.05).

**Conclusions:**

Arthroscopic rotator cuff suture and simultaneous 360° capsular release have a significant effect on the treatment of rotator cuff tear with limited shoulder movement.

## Background

Rotator cuff tear is one of the important factors affecting shoulder function. Conservative treatment is often used in early treatment, but the recovery process is long and difficult, and does not meet the clinical and psychological needs of patients [1]. Therefore, rotator cuff injuries have become a focus of research in an attempt to develop treatment methods with a good curative effect, a short healing time, and a high healing rate [1]. With the development of arthroscopic technology, arthroscopic suturing of the rotator cuff has become the main method with which to treat rotator cuff injuries [2]. The simultaneous capsular release and rotator cuff sutures has been demonstrated to have a significant impact on the treatment of rotator cuff injuries, although the manipulation is uncontrollable and frequently results in other forms of injuries [3]. Arthroscopic capsular release is dependable, safe, and has less problems [4]. Another study showed that a 360° capsular release of the joint capsule with simultaneous arthroscopic suturing is more effective [5]. Based on this finding, the current study retrospectively analyzed the effects of rotator cuff suturing and arthroscopic 360° capsular release on the Constant-Murley and Visual Analogue Scale (VAS) scores, and shoulder flexion in patients with rotator cuff injuries. The combination of two different surgical approaches was then studied to determine the clinical efficacy of rotator cuff suturing and arthroscopic 360° capsular release in patients with rotator cuff tears and limited shoulder motion.

## Methods

### General information

Fifty-one patients (16 males and 35 females) with severe rotator cuff injuries and limited shoulder movement (17 left and 34 right shoulders) who were admitted to our hospital from October 2017 to October 2020 were selected as study subjects. The patient age range was 39–72 years, with an average age of 57.00 ± 2.24 years. The duration of disease ranged 6–25 months, with an average disease duration of 15.50 ± 0.29 months. All patients had severe joint pain, mainly at night, with limited movement, and the flexion angle ranged from 35°-68°, with an average angle of 51.50 ± 2.10°. All patients failed conservative treatment for at least 3 months before surgery.Criteria for patients to undergo surgery are based on the judgment of a specialized surgeon.The study was approved by the Ethics Committee of our hospital and all patients voluntarily signed an informed consent form.

### Diagnosis, and inclusion and exclusion criteria

The diagnostic criteria for a rotator cuff tendinopathy were as follows: (1) the rotator cuff tear was diagnosed by MRI examination and verified by physical examination; (2) the age of ≥18; (3) the patient and their family had understood the study and agreed to operate for the return and follow-up; (4) the patient had complete clinical data; (5) the shoulder joint function was normal before the onset; (6) Patients with rotator cuff tears and global stiff shoulder with limited shoulder motion (7) the full layer of the supraspinatus tear, and the tear size was [4].

The inclusion criteria were as follows: no obvious history of trauma.

The exclusion criteria were as follows: (1) Combined shoulder fracture, significant glenoid labral injury of the shoulder joint, glenoid labral repair and fixation, need to cut or fixate the long head tendon of the biceps muscle (2) Tear range of > 5cm (massive rotator cuff tears); (3) Previous history of shoulder surgery; (4) accompanied by the dominant nerve injury of the affected limb; (5) Age is <18 years old; (6) Patients with severe shoulder joint infection, tumor and other diseases; (7) Patients who have serious other physical diseases and cannot tolerate surgery; (8) Patients with communication difficulties due to cognitive impairment or poor language ability.

### Treatment

Lying on the side under general anesthesia, the arthroscope was first introduced from the rear to explore the internal tissues of the rotator cuff, including the joint capsule, synovium, biceps tendon, and the articular surface of the rotator cuff. Then, from anterior and bilateral approaches, a 360° capsular release was performed. The anterior, posterior, superior, and inferior joint capsules and the glenohumeral ligament were alternately released alternately, the rotator cuff interval was cleared, and the coracoid process was formed. Other anomalies were addressed in turn. Then, the subacromial bursa was cleaned and an acromioplasty was performed to turn the hooked acromion into a flattened acromion. An anchor was positioned on the footprint bone’s surface at the margin of the joint to suture tendons in joints. The rip was sutured from back to front through the rotator cuff tendon following complete relaxation of the rotator cuff tendon. As a bone marrow-stimulating (BMS) technique for rotator cuff healing, the microfracture was performed before repair on the greater tuberosity.

Within 24 h of surgery, the patient recovered in bed, elevating the injured limb, and periodically applying ice to the surgical site. Mild passive joint exercises were started on the first postoperative day and continued for four weeks before muscle strength training was gradually introduced.

### Observation indicators

All patients were treated with arthroscopic rotator cuff suturing and 360° capsular release surgery. The Constant-Murley and VAS scores were used before and during the postoperative follow-up period, respectively, and the shoulder flexion angle was recorded. The Constant-Murley score was evaluated as follows: joint activity (5 points); daily social activities (3 points); and muscle strength (2 points). The VAS score was categorized as follows: severe pain, 9–10 points; general pain, 6–8 points; mild pain, 3–5 points; and no pain, 0–2 points. One year after the operation, recovery was assessed by magnetic resonance imaging (MRI). According to the Sugaya classification, rotator cuff healing was divided into 5 categories: category I, low signal and sufficient thickness of the rotator cuff; category II, adequate rotator cuff thickness, but there is a high signal; category III, the thickness of the rotator cuff layer is < 50% of the normal tendon, but there is no discontinuity of the tendon; category IV, there are 1–2 images showing the continuity of the tendon; and category V, the presence of more than 2 tomographic images suggests a discontinuity of tendon continuity. Categories I ~ III are regarded as clinical healing.

### Statistical methods

The data were analyzed using SPSS18.0 statistical software. Measurement data are described as the $\overline x \pm s$, and comparisons were made using a paired sample t-test. Count data are described as the percentage (%), and comparisons were made using a χ2 test. P < 0.05 indicated that the difference was statistically significant.

## Results

### Constant-murley score

The Constant-Murley score after surgery was significantly higher than before surgery (P < 0.05), as shown in Table 1.

**Table 1:** Comparison of Constant-Murley score ($\overline x \pm s$, score)

Group	Before operation	After operation	t	P
n	51	51	-	-
Subjective part (score)	10.35±4.64	19.47±5.88	13.143	0.000
*Objective part (score)*	8.98±6.69	39.51±6.61	17.936	0.000
*Totals (score)*	29.33±9.71	58.98±9.84	19.696	0.000

### Shoulder flexion angle and VAS score

The postoperative shoulder flexion angle was significantly improved compared with the preoperative shoulder flexion angle (P < 0.05). The VAS score was significantly lower than before surgery (P < 0.05), as shown in Table 2.

**Table 2:** Comparison of shoulder flexion angles and VAS score ($\overline x \pm s$)

Group	Before operation	After operation	*t*	*P*
n	51	51	-	-
Shoulder flexion angle(°)	51.50 ± 2.10	142.67 ± 8.59	73.627	0.000
VAS score (score)	7.54 ± 1.22	1.23 ± 0.87	30.073	0.000

### Rotator cuff healing

Based on a review of MRI technology 1 year postoperatively (October 2020), the number of indicators for the Sugaya classification is shown in Table 3.

**Table 3:** Number of Sugaya classified indicators (person)

Group	Category I	Category II	Category III	Category IV	Category V
Before operation	0	0	0	0	51
After operation	6	43	1	1	0

## Discussion

There are many theories regarding the pathogenesis of rotator cuff injury, with the most common being external impingement, internal impingement, and tendons intrinsic degeneration. External impingement includes subacromial impingement and subcoracoid impingement, while internal impingement involves entrapment of the posterosuperior rotator cuff tendons between the humeral head and posterior glenoid during abduction and external rotation. The cause of tendons intrinsic degeneration is generally consider to be the consequence of overuse [6]. Arthroscopic rotator cuff suture and simultaneous 360° capsular release have a significant effect on the treatment of rotator cuff tear with limited shoulder movement [7]. The initial pain that accompanies a rotator cuff tendinopathy is not obvious in the case of a mild injury, while severe pain occurs immediately in severe cases. The clinical manifestations are night pain, back and hand pain, and “pain arc”. Shoulder joint movement is limited, and the free abduction and forward flexion cannot be performed, and serious obstacles to life [8]. Due to a lack of accurate diagnostic methods in the early stage, misdiagnosis frequently occurs and the optimal treatment time is delayed. In 1931, Codman [9] was the first to systematically describe the manifestations of rotator cuff injuries and proposed diagnostic and surgical methods for each manifestation. Early treatment of rotator cuff injuries is mostly conservative, with surgery following recovery of passive motion or failure of conservative treatment [10]. Research has confirmed that conservative treatment can alleviate symptoms of rotator cuff tear, but cannot improve range of motion in the long term [1]. The basis for this finding is that manual release has little effect on improving the range of motion of the glenohumeral joint, unlike the relative range of motion between the scapula and chest wall. Conservative treatment has limitations. In fact, although the potential complications of surgical treatment (such as postoperative stiffness, infection) cannot be ignored, conservative treatment cannot restore the tendon, which increases the risk of shoulder tendon degeneration over time [11]. Depending on patient needs, MRCT surgical treatment may have different goals and there are different arthroscopic approaches to address the problem. Debridement and long head of the biceps tenotomy or tenodesis have been used in patients with less demanding conditions where the main symptom is pain and shoulder function is sufficient for their activities of daily living [12]. Tuberoplasty [13] and “insertion techniques” such as subacromial balloon [14] and superior capsule reconstruction (SRC) [15] are designed to relieve pain and improve function by facilitating subacromial slide of the humeral head, lower the humerus head. Arthroscopic repair or partial repair can improve function and control pain.

With the rapid development of medical technology, the update of imaging instruments (color Doppler, CT, and MRI) has improved the diagnostic accuracy of rotator cuff injuries, and the emergence of arthroscopy has compensated for the shortcomings of traditional diagnosis and treatment methods.Arthroscopic rotator cuff repair surgery is an effective means to solve the above problems. Arthroscopic rotator cuff suture, as a surgical method to repair the torn rotator cuff, achieves mechanical balance, repairs acromion impingement, and plays an important role in the treatment of rotator cuff injuries. Since the 1990s, many surgeons have proposed arthroscopic minimally invasive techniques. This method has become a common choice for patients with rotator cuff injuries after conservative treatment fails. Early arthroscopy was only used for simple debridement of lesions, and major intra-articular surgeries can now be performed using an arthroscope [16].

There are three ranges of arthroscopic release: 90°; 270°; and 360°. Arthroscopic lateral supine 360 degree joint capsule release surgery for the treatment of idiopathic joint capsule adhesion can significantly improve the range of motion in an early and persistent manner, achieve good functional results, and reduce revision and complication rates [17]. The importance of improving shoulder function is explained in detail. This surgical method can be performed only under arthroscopy, without the need to separate the deltoid muscle or make a large incision on the body. Brenneke et al. found that the shoulder capsular tissues in different positions help to stabilize the shoulder in different orientation [18], which may contribute to the stiffness of the shoulder. Ma and other studies also have shown that patients with rotator cuff injuries can greatly improve symptoms after rotator cuff suturing and arthroscopic 360° capsular release treatment [7]. Our study also showed that after rotator cuff suturing and arthroscopic 360° capsular release in patients with a rotator cuff tear, the Constant-Murley score was significantly increased, the VAS score was significantly decreased, the shoulder flexion angle was significantly increased, and postoperative clinical healing achieved a high rate. However, capsular release can cause postoperative micro-instability, and the lack of evaluation of long-term functional and radiological outcomes is a limitation.

This finding shows that rotator cuff suturing and arthroscopic 360° capsular release have a significant effect on the treatment of patients with a rotator cuff tear with limited shoulder movement.

## Conclusions

In conclusion, arthroscopic rotator cuff suture and simultaneous 360° capsular release have a significant effect on the treatment of rotator cuff tear with limited shoulder movement, which is worthy of clinical promotion.

## Data Availability

The datasets used and/or analysed during the current study are available from the corresponding author on reasonable request.

## Abbreviations

VAS: Visual Analogue Scale
MRI: Magnetic resonance imaging
